# Ankylosing spondylitis: etiology, pathogenesis, and treatments

**DOI:** 10.1038/s41413-019-0057-8

**Published:** 2019-08-05

**Authors:** Wei Zhu, Xuxia He, Kaiyuan Cheng, Linjie Zhang, Di Chen, Xiao Wang, Guixing Qiu, Xu Cao, Xisheng Weng

**Affiliations:** 1 Department of Orthopedics, Peking Union Medical College Hospital, Chinese Academy of Medical Sciences & Peking Union Medical College, 100730 Beijing, China; 2 Department of Clinical Medicine, Chinese Academy of Medical Sciences & Peking Union Medical College, 100730 Beijing, China; 3 Department of Orthopedic Surgery, Rush University Medical Center, Chicago, IL 60612 USA; 4 Department of Orthopedic Surgery, School of Medicine, Johns Hopkins University, Baltimore, MD USA

**Keywords:** Calcium and phosphate metabolic disorders, Pathogenesis

## Abstract

Ankylosing spondylitis (AS), a common type of spondyloarthropathy, is a chronic inflammatory autoimmune disease that mainly affects spine joints, causing severe, chronic pain; additionally, in more advanced cases, it can cause spine fusion. Significant progress in its pathophysiology and treatment has been achieved in the last decade. Immune cells and innate cytokines have been suggested to be crucial in the pathogenesis of AS, especially human leukocyte antigen (HLA)‑B27 and the interleukin‑23/17 axis. However, the pathogenesis of AS remains unclear. The current study reviewed the etiology and pathogenesis of AS, including genome-wide association studies and cytokine pathways. This study also summarized the current pharmaceutical and surgical treatment with a discussion of future potential therapies.

## Introduction

Spondyloarthropathy (SpA) refers to a heterogeneous group of rheumatic diseases that present common clinical and genetic features, which are classified as peripheral or axial (axSpA) based on what parts of the body are predominantly affected. Ankylosing spondylitis (AS), a type of SpA, is an autoimmune disease that mainly involves spine joints, sacroiliac joints (SIJs) and their adjacent soft tissues, such as tendons and ligaments. In more advanced cases, this inflammation can lead to fibrosis and calcification, resulting in the loss of flexibility and the fusion of the spine, resembling “bamboo” with an immobile position. The main clinical manifestations include back pain and progressive spinal rigidity as well as inflammation of the hips, shoulders, peripheral joints and fingers/toes. In addition, there are extra-articular manifestations, such as acute anterior uveitis and inflammatory bowel disease (IBD). However, these extra-articular manifestations differ between East Asian and Caucasian populations. In a study involving 988 patients with ankylosing spondylitis in east Asia, only 0.4% developed inflammatory bowel disease.^1^ However, in some analyses performed in Western countries, ~5%–10% of patients with AS present with inflammatory bowel disease.^2,3^

The prevalence of AS has a clear correlation with the human leukocyte antigen (HLA)-B27 positive rate in specific populations. Studies have revealed that in HLA-B27-positive populations, the prevalence rate of AS is ~5%–6%.^4^ In a 2009 national survey in the United States, the prevalence of HLA-B27-positive populations varied in different ethnic communities, with 7.5%, 4.6%, and 1.1% in non-Hispanic whites, Mexican-Americans, and non-Hispanic blacks, respectively.^5^ In the literature, males reportedly account for the vast majority of cases of AS, while the incidence among men and women is similar in nonradiographic axial spondyloarthropathy (nr-axSpA), which refers to individuals meeting clinical criteria for axSpA without radiological evidence of sacroiliitis. A meta-analysis including eight studies including 2 236 patients with AS and 1 242 patients with nr-axSpA revealed that males accounted for 70.4% of AS patients and 46.5% of patients with nr-axSpA.^6^ Genetic susceptibility results have shown the following recurrent risk factors in different generations of relatives: monozygotic (MZ) twins, 63% (17/27); first-generation relatives, 8.2% (441/5 390); second-generation relatives, 1.0% (8/834); and third-generation relatives, 0.7% (7/997).^7^

Available criteria sets are frequently used in clinical practice to help clinicians make diagnoses. Currently, the most widely applied diagnostic classification of AS is the modified New York (mNY) criteria. In this classification system, a patient needs to meet at least one clinical criterion and the radiological diagnosis of AS. Another classification is Amor criteria and European Spondyloarthropathy Study Group (ESSG) criteria for diagnosing AS. In 2012, AV Tubergen discussed the different classification criteria sets for AS and SpA.^8^ In addition, the ASAS criteria, the dominant diagnostic criteria for axSpA, have gained popularity in Europe (2016 update of the ASAS-EULAR management recommendations for axial spondyloarthritis). As clinicians seemed to have difficulty differentiating AS and SpA, Joel D. Taurog established an algorithm for the diagnosis or exclusion of axSpA.^9^

The confusion in diagnosis and lack of disease-modifying therapeutics, including anti-TNF-α and anti-IL-17 treatment of AS, are largely due to the limited knowledge of the pathogenesis, which may involve immunity, heredity and other factors. In this paper, we reviewed the etiology of AS, current investigations of its pathogenesis and available treatments.

### Etiology

As an autoimmune disease, AS develops through complex interactions between genetic background and environmental factors. Although significant progress has been achieved in the past decades, the etiology of AS remains unclear to some extent. To date, studies have revealed some factors that may be related to the occurrence of AS, including genetic background, immune reaction, microbial infection, and endocrinal abnormity.

### Genetic background

Genetic factors have been acknowledged as crucial in the genesis of AS. The correlation between AS and genetics has been a perpetual topic since hereditary factors of AS were first confirmed within families in 1961.^10^ Twin studies have revealed significantly higher concordance between monozygotic twins (63%) than between dizygotic twins (23%). Genetic effects have been identified as pathogenic factors that contribute to over 90% of the population variance for AS manifestations.^11,12^ One of the most important genetic factors is major histocompatibility complex (MHC) class I allele HLA-B27, which was discovered in 1973.^13^ Despite the unclear pathomechanism, HLA-B27 has been associated with the prevalence of AS in different populations around the world.^14^ Studies have shown that 90%–95% of AS patients are HLA-B27 positive, while 1%–2% of HLA-B27-positive populations develop AS. This number increased to 15%–20% for those with an affected first-degree relative.^15,16^ The familial tendency of AS was remarkable with relative risks of 94, 25, and 4 for first-, second-, and third-degree relatives, respectively.^17^ In addition to the association with the genesis of AS, HLA-B27-positive patients showed a significantly lower average onset age and a higher prevalence of acute anterior uveitis than did HLA-B27-negative patients.^18^ HLA-B27 has a high degree of polymorphism. Over 100 subtypes have been identified thus far,^19^ with differing prevalence rates among different ethnicities, especially between those of East Asian and Caucasian descent. As reported, the most prevalent subtypes in AS are HLA-B2705 (Caucasian populations), HLA-B2704 (Chinese populations), and HLA-B2702 (Mediterranean populations).^19–22^ By contrast, two subtypes, HLA-B2706 and HLA-B2709, seem unrelated to AS.^17,23,24^ In addition, genetic influence is not alone in the development of AS. HLA-B27-transgenic rat studies on β2 microglobulin (β2m),^25^ a noncovalent part of the MHC-I complex, has proven that additional β2m reduces HLA-B27 misfolding and promotes arthritis and spondylitis, implying that B27 misfolding is associated with intestinal inflammation.^26^ This result suggested that abnormal β2m can coordinate with HLA-B27 in AS development, which may be explained by protein misfolding theories and will be discussed later in the pathogenesis section.

Even as the most emphasized genetic factor, the overall contribution of HLA-B27 to AS heritability is only ~20%,^27^ indicating that other genetic influences contribute to AS. With the progress of genome-wide association studies (GWASs) and other technologies, non-HLA-B27 and even non-HLA genes have been identified in AS in recent years; basically, the genetic differences between various ethnicities have been explored and compared in recent years. HLA-B60 is related to HLA-B27-negative AS and increases the disease susceptibility by 3–6-fold.^28^ An analysis in a Taiwanese population suggested that the interaction between HLA-B60 and HLA-B27 could be a better marker for the risk of AS susceptibility.^29^ HLA-B7,^30^ HLA-B16, HLA-B35,^31,32^ HLA-B38 and HLA-B39^33^ have also been associated with HLA-B27-negative AS in various ethnicities with unknown mechanisms. A study of 1 000 AS patients and 1 500 heathy individuals reported an initial association and independent replication of two new loci related to AS, *ERAP1* (also known as *ARTS1*) and *IL23R*, in a North American sample and provided preliminary evidence for several non-MHC nonsynonymous single nucleotide polymorphisms (nsSNPs).^34^ Another study including GWAS of 2 053 AS patients and 5 140 controls presented SNPs in two gene deserts at 2p15 [rs10865331; combined *P* = 1.9 × 10(−19)] and 21q22 [rs2242944; *P* = 8.3 × 10(−20)], as well as in the genes *ANTXR2* [rs4333130; *P* = 9.3 × 10(−8)] and *IL1R2* [rs2310173; *P* = 4.8 × 10(−7)].^35^ Many GWASs have been performed in Caucasian and East Asian populations, focusing on genetic polymorphisms of AS susceptibility. One of these studies^36^ genotyped 69 non-MHC AS-associated SNPs and found that six loci (rs6759298, rs2297518, rs75301646, rs12615545, rs5837881, and rs27044) showed significant differences in both Caucasian and EA populations, with rs6759298 located in a ‘gene desert’ at chromosome 2p15 exhibiting the highest significance. However, genetic differences in several SNPs have been identified. For example, rs27980 and rs7711564 in *ERAP1* previously identified in Caucasian descent were significantly associated with AS in Han Chinese, while no association was shown for *ERAP1* SNPs such as rs27044.^37–39^ Similarly, *IL23R* (rs11209026), widely considered a major AS-associated SNP in Europe, showed no significance in a Chinese AS population.^37^ These studies on non-HLA-B27 genes and SNPs indicated a difference in the mechanism of disease pathogenesis between various ethnicities and may provide new insights into the pathogenesis and treatment of AS.

### Immunological and microbial factors

AS is related to a series of autoimmune diseases, including IBD, anterior uveitis and psoriasis, which suggests that they may share a genetic basis and some common immunological processes. The differences observed in immune cells and cytokines in AS suggest the role of immunological effects in AS pathogenesis. In the peripheral blood of AS patients and healthy HLA-B27-positive controls, the levels of T cells secreting tumor necrosis factor (TNF)-α and interferon (IFN)-γ were reportedly lower. CD8+ T cells in AS patients tended to secrete more IL-10.^40^ Other findings have also demonstrated immunological influences in AS development, which is discussed in the following section.

Microbial infection acts as a triggering factor of the host innate immune system and AS development.^41^ HLA-B27 transgenic rats failed to develop features of SpA in a germ-free environment, which changed when commensal bacteria were introduced into the germ-free models,^42,43^ suggesting possible interactions between HLA-B27 and the microbiome. The gut microbiome, including *Lachnospiraceae*, *Veillonellaceae*, *Prevotellaceae*, *Porphyromonadaceae*, and *Bacteroidaceae*, showed significant differences in AS patients compared with that in healthy controls.^44^ *Klebsiella pneumoniae* acts as an opportunistic pathogen in the normal human gut, and studies have suggested that it may be an exacerbating agent in the autoimmune process of AS.^45^ Controversial results exist regarding the relationship between the fecal microbiome load, such as *Klebsiella pneumoniae*, and AS activity. Some scientists hypothesized that *Klebsiella pneumoniae* influences AS development indirectly through interplay with HLA-B27.^46^ In addition, gut microbiome infection is partly due to the relative deficiency of immune components, leading to immune responses of a higher intensity and longer duration.^47^

### Other factors

Early in 1973, an etiological association between endocrine factors and AS was hypothesized because the presence of HLA-B27 and AS differed with sex.^48^ A study of 22 patients with AS detected testicular function and found a diminished testicular testosterone (T) reserve, elevated luteinizing hormone (LH) level, estradiol/testosterone ratio (E2:T) inversion and slightly increased estradiol (E2) level.^49^ The results in studies of ovarian function have also indicated sex hormone differences in menstruating and menopausal AS patients compared with those in matched healthy controls.^50^ It is reported that estradiol levels in patients with active AS are significantly lower than those in patients with inactive AS in the menstruation period. More observational results, such as male predominance, peak onset at young age and increased number of first manifestations after pregnancy, imply that sex hormones play a role in AS.^51^ Low levels of sex hormones, especially dehydroepiandrosterone sulfate (DHEAS), may also contribute to bone loss in patients with AS.^52^ A study examining the low-dose adrenocorticotropic hormone (ACTH) test (LDST) showed that after low-dose ACTH, the cortisol increment was significantly lower in AS patients than in controls (20.0 ± 4.4 vs 24 ± 2.2 microg/dl, *P* < 0.001).^53^ The subclinical glucocorticoid deficiency indicated an impaired hypothalamic-pituitary-adrenal (HPA) axis in AS patients, suggesting the involvement of the endocrine system in the etiology of AS.

Meta-analyses have suggested that vitamin D deficiency may be related to AS development. Vitamin D may play a protective role in AS based on a positive correlation between the serum vitamin D level and disease severity.^54^ There is a significant negative correlation between the vitamin D level and disease activity indicated by the Bath Ankylosing Spondylitis Disease Activity Index (BASDAI), erythrocyte sedimentation rate (ESR) and C-reactive protein (CRP) level.^55^ However, there are conflicts regarding the relationship between the vitamin D level and AS disease activity.^55,56^ The cause of low vitamin D levels in patients with AS is also largely unclear.

## Pathogenesis

### MHC genetics

The human MHC, also called the HLA complex, belongs to the cell-surface proteins acting in the process of acquired immunity. There are three subgroups in the MHC gene family: class I, II, and III. MHC class I encodes HLA-A, HLA-B, and HLA-C and is present on all nucleated human cells and platelets, presenting epitopes to T cell receptors (TCRs) on the surface of cytotoxic T lymphocytes (CTLs).^57^ The heterodimer MHC class I subgroup consists of a polymorphic heavy chain. The chain contains three domains, i.e., α1, α2, and α3. The α1 domain links noncovalently with the non-MHC molecule β2m, while α3 spans the plasma membrane and interacts with the CD8 coreceptor of T cells.^58,59^ The MHC class I complex can link to peptides of 8–10 amino acids in length via one cleft spaced by both α1 and α2, leading to the initiation and propagation of immune responses.^59,60^ A stable MHC molecule needs to be properly packaged and then folded in the cell organelle endoplasmic reticulum (ER) under guidance of chaperones (calreticulin and tapasin).^57^ Although the classic MHC class I contains one heavy chain, there are three different structures of MHC‐I, comprising cell-surface HLA‐B27 homodimers and intracellular and exosomal MHC‐I dimers.^61^ These components may function in distinct pathophysiological processes.

### HLA-B27

HLA-B27, basically belonging to the MHC-I surface protein encoded by the MHC B gene on chromosome 6, is the most essential gene that predisposes an individual to AS. HLA-B27 presents peptide antigens to T immunocytes of the human body defense process and is considered to be significantly linked to AS and associated inflammatory diseases. A study reviewed over 7500 endogenous peptides presented by the eight most frequent HLA–B27 allotypes (HLA–B2702 to HLA–B2709), suggesting that consensus-binding and selection motifs showed significant similarities and differences between various HLA–B27 allotypes.^62^ The connection between HLA-B27 and AS has not yet been fully elucidated, although it is widely accepted that the entire intracellular process of HLA-B27 formation needs to be considered. There are some prevailing theories regarding the mechanism, including the hypothesis of arthritogenic peptide, misfolding hypothesis, the hypothesis of molecular mimicry, as well as the hypothesis of the cell-surface HLA‐B27 homodimer.

Founded on the antigenic peptide presentation role of HLA class I molecules, the arthritogenic peptide hypothesis postulates that structurally exclusive peptide‐MHC complexes can directly initiate HLA‐B27-specific autoimmune responses by relying on the primary structure of antigen peptides.^63^ Some microbial peptides are similar to self-peptides in body tissues and can activate the response of certain HLA-B27-specific CD8+ T lymphocytes. The T lymphocytes react with these HLA-B27-peptide complexes, leading to autoreactivity and autoimmune disease.^64,65^ It has been suggested that cartilage, particularly the proteoglycan aggrecan,^66^ is the basic immunological target in SpA; however, currently, studies on such peptides have obtained inconsistent results. In addition, rats with HLA-B27-specific CD8- T lymphocytes still have AS, which means that more peptide mechanisms remain unelucidated.^67^ Additionally, a spectrometry study reviewed a large quantity of HLA-B27 subtypes and indicated that it is quantitative instead of qualitative changes in the peptide repertoire that may be more relevant to AS initiation and progression,^62^ further challenging the arthritogenic peptide hypothesis. The molecular mimicry hypothesis posits that the antigenic components of infectious bacterial pathogens partially resembling or cross-reacting with HLA molecules can stimulate CD8+ T lymphocytes, followed by responding to one HLA‐B27 relevant self‐peptide or the peptides directly produced by HLA‐B27.^68^ This hypothesis is largely based on previously identified amino acid structures of homologous origin between the HLA structure and specific sequences and previous results depicting cross-reactions among the HLA and some bacterial antigens.^46^ *K. pneumoniae* is a highlighted microorganism thought to participate in the pathogenesis of AS as a triggering and/or perpetuating factor.^69^ Some components in *K. pneumoniae* share structural likenesses with specific genetic or somatic sequences in humans and exhibit molecular mimicry in AS and other diseases. Similarly, molecular modeling suggested that a HLA-B27-derived dodecamer, a natural ligand of disease-associated B27 subtypes, was strikingly homologous to protein sequences from arthritogenic bacteria, particularly *Chlamydia trachomatis*, demonstrating the process of molecular mimicry of Chlamydial proteins.^70^ PulD-secreted pullulanase can cross-react with HLA-B27 and myosin, while pulA components can cross-react with type I, III, and IV collagens,^46^ proving the reasonability of the molecular mimicry hypothesis. These cross-reactions give rise to an amount of antibacterial antibodies that link to HLA molecules on immunocytes, chondrocytes and fibroblasts,^71^ further triggering a cascade of inflammatory reactions with the amount of cytokines, complement proteins, proteinases and the like produced.^72^ These sequential reactions lead to the genesis of arthritis and extra-articular or even systemic symptoms and signs of AS.

The mature HLA-B27 complex is a quaternary structure with three important components. The proper assembly and folding of HLA-B27 in the ER is essential for its function. After being synthesized as free heavy chains, HLA-B27 is then noncovalently linked and folded with β2m and antigenic peptide, followed by transport to the cell surface as a trimolecular complex.^73^ Nevertheless, HLA‐B27 exhibits a predisposition to misfolding and creating dimers and even multimers^74^; these characteristic changes may originate in its structure, which includes cysteine (C) at sites 67 (C67), 101 (C101), 164 (C164), and 325 (C325).^75^ Without correct folding, HLA-B27 would be produced and transmitted to the cell surface merely as homodimers consisting of heavy chains. The disease‐related structures of HLA‐B27, including HLA‐B2705, HLA‐B2704, and HLA‐B2702, have been found to exhibit a relatively lower rate of correct folding procedures compared with those of HLA‐B2706 and HLA‐B2709, which are generally not considered associated with AS.^76^ Due to cysteine residue C67 and other reasons, HLA-B27 tends to fold slower than other HLA alleles, and without proper folding, these defective HLA-B27 proteins continually gather in the ER.^73^ Improperly folded HLA-B27 proteins accumulate in the ER and activate autophagy and the interleukin (IL)-23/IL-17 pathway.^73^ Moreover, these misfolded molecules can interfere with ER function, leading to ER stress and even triggering the pro-inflammatory endoplasmic reticulum unfolded protein response (ERUPR), which further activates the IL-23/IL-17 pathway.^73,77^ However, conflicts also exist regarding whether the HLA-B27-activated ERUPR occurs in AS patients. The increased production of IL‐23 without significant ERUPR induction occurs in macrophages in AS.^78^ The disease‐related polymorphisms of the ERAP1 or HLA‐B27 locus would not change the ER stress intensities of AS,^79^ which also remained controversial in other studies conducted later.^14^ One possibility is that HLA-B27 misfolding results in autophagy and triggers the IL-23/IL-17 pathway instead of ERUPR.^80^ Further research is required for illumination of the connection of ERUPR and HLA-B27 during the development of AS.

**Fig. 1:**
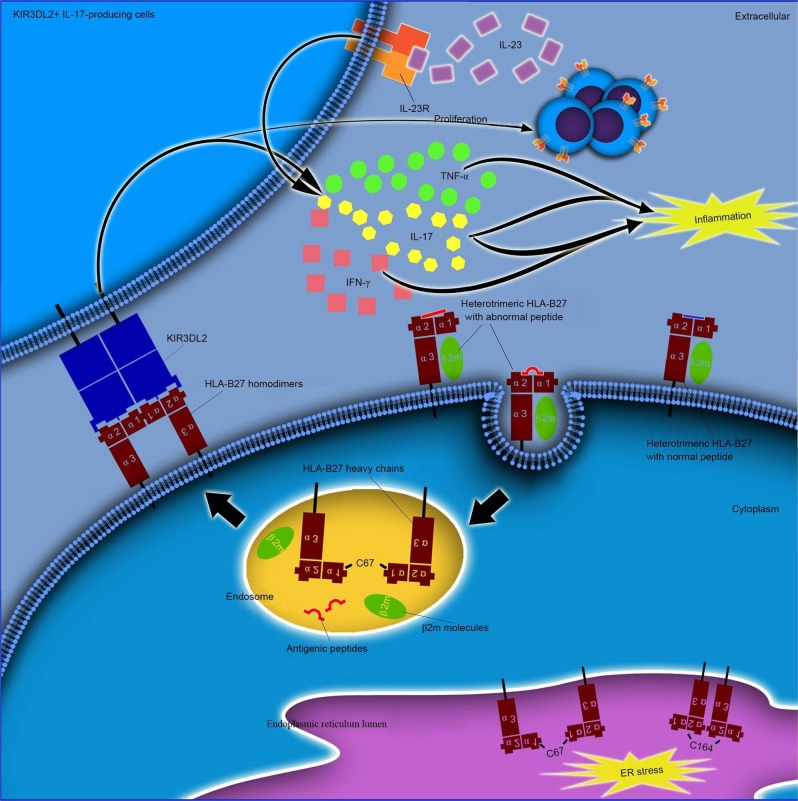
Various functions of ER resident and cell surface HLA-B27 dimers. ER resident dimers might lead to ER stress and activate unfolded protein responses. Cell-surface dimers might be produced after the recycling of fully folded HLA-B27 cell surface molecules through the endocytic pathway and be re-expressed as dimers for presentation to receptors, such as KIR and LILR. Elevated propagation and existence of KIR3DL2+ CD4+ T lymphocytes and amplified IL-17 production in AS cases after stimulation with antigen-presenting cells expressing HLA-B27 homodimers were confirmed in earlier studies. These cells have largely been revealed to secrete TNF-α and IFN-γ. IL-17 has been shown to coordinate with TNF-α or IFN-γ in stimulating the release of inflammatory regulators and affecting bone construction to function in the development of AS. (Reprinted with permission of Elsevier, from chen et al.^75^)

HLA-B27 heavy chains tend to form homodimers without β2m via the disulfide bonds of the cysteine at C67.^81^ The dimeric HLA-B27 complexes, mostly found in the gut and synovium of patients, may contribute to the genesis of AS and some other SpAs. These HLA-B27 dimers could occur on antigen-presenting cells, thus stimulating IL-23 receptor + T lymphocytes to produce IL-17.^82^ The hypothesis of cell-surface HLA‐B27 homodimer formation suggests that HLA-B27 dimers might contribute to the development of AS. HLA‐B27 homodimers have been linked to receptors expressed on natural killer (NK) immunocytes, myelomonocytes and lymphocytes. The binding is realized via killer cell immunoglobulin‐like receptors (KIRs) and leucocyte immunoglobulin‐like receptors (LILRs), thus acting in the processes related to autoimmune disorders (Fig. 1).^83,84^ The 3 immunoglobulin domains and the long cytoplasmic tail 2 (KIR3DL2) receptor expressed by certain increased immune cells, including NK cells and Th17 cells, can recognize cell-surface HLA‐B27 homodimers via a greater affinity than that with the classic HLA‐B27 heterotrimers.^85,86^ The binding of KIR3DL2 with HLA-B27 homodimers was revealed to stimulate the survival and differentiation of KIR3DL2+CD4+ T lymphocytes in patients with SpA.^87,88^ Compared to KIR3DL2− lymphocytes, these T cells significantly increase cytokine output, including IL‐17, TNF‐α and INF-γ.^85^ These findings suggest that the aberrant HLA‐B27 homodimers function in AS pathogenesis.

### Non-HLA-B27 MHC alleles

In addition to the widely accepted HLA-B27 genetics in AS, other non-HLA-B27 MHC alleles, including multiple MHC-I or II loci, have been associated with the pathogenesis of AS. These genes, including HLA-B40, HLA-B60, HLA-A, HLA-DRB1, HLA-DQA1, HLA-DPB1, or others, function in the development of AS via interaction with TCRs and KIRs expressed on NK cells and certain lymphocytes or participate in antigen presentation and other inflammatory processes.^14^ For example, HLA-G was proven to produce homodimers in the cell organelle endosomes through a completely folded β2m‐related form.^89^ Non-HLA-B27 genes may act independently or via linkage disequilibrium with HLA-B27. Additionally, HLA-A0201 tag SNP rs2394250 is reportedly related to AS independent of HLA-B27 for HLA-B27-positive or HLA-B27-negative AS patients.^90^ The mechanisms by which these non-HLA-B27 MHC genes affect AS remain largely unknown. Further study on the non-HLA-B27 MHC allele may offer insights into the pathogenesis of SpA and other related diseases.

### Non-MHC genetics

In the past four decades, research on the pathogenesis of AS has been focused on MHC genes. However, the development of GWAS methods and the identification of a considerable number of genetic variants revealed the importance of non-MHC genes. Although the overall contribution of HLA-B27 is only ~20%, making HLA-B27 a major contributor, ~7% of the heritable risk originates from non-MHC variants.^27,91,92^ Therefore, non-MHC genetics also function in the pathogenesis of AS. Moreover, some pathways or mechanisms have been reported to be involved in the processes of AS in recent years.

### ERAP1 and ERAP2

Three previously identified aminopeptidases were recognized as genetically related to AS vulnerability, including *ERAP1* (coding for endoplasmic reticulum aminopeptidase 1 (ERAP1)), *ERAP2* (coding for ERAP2), and *NPEPPS* (coding for puromycin-sensitive aminopeptidase (PSA)).^90,93^ Recent research has indicated that gene-to-gene interactions among HLA-B27 and *ERAP1* and subsequent abnormal peptide presentation are likely relevant to the development of AS (Fig. 1).^90,94^ A case-control association study revealed that protective genetic variants were associated with reduced function of *ERAP1* and *ERAP2* and suppressed MHC-I expression on the cell surface.^90^ *ERAP1* and *ERAP2* variations may also reduce the speed of HLA-B27 folding by affecting the amount of relevant peptide accessible, thereby increasing ER stress and AS development.^14^

Both ERAP1 and ERAP2, genes at chromosome 5q15, participate in trimming peptides in the ER to nine amino acids for antigen presentation by HLA-I molecules, such as HLA-B27.^95^ In addition to the processing and presentation of antigens, ERAP1 can still trim several cytokine receptors on the cell surface, such as IL-1R2, TNFR1, and IL-6Rα, thus reducing their ability to conduct signals to cells, and the latter further affects inflammatory processes.^96^ These two genes are involved in the development of AS and other diseases. ERAP1 is reportedly associated with HLA‐B27− and HLA‐B40­positive AS,^95^ while ERAP2 is related to HLA‐B27+ and HLA‐B27− AS.^97^ ERAP1 is also involved in the development of juvenile idiopathic arthritis, psoriasis, and Behçet’s disease, while ERAP2 is related to Crohn’s disease and psoriasis, as well as birdshot chorioretinopathy,^79,91^ with unclear mechanisms.

**Fig. 2:**
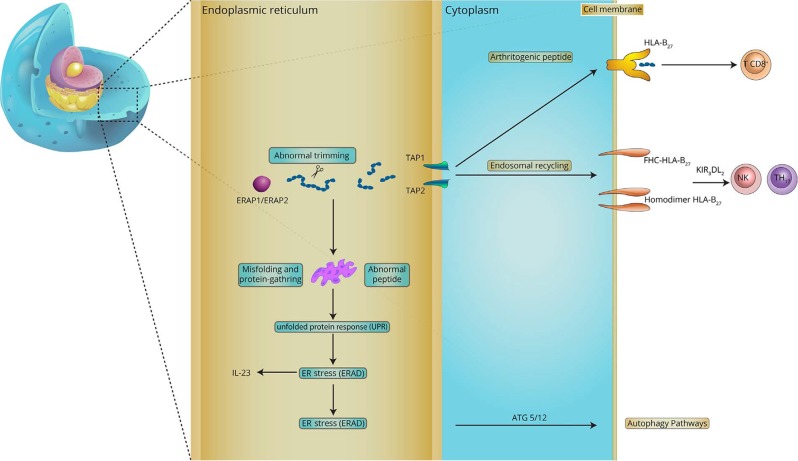
Demonstration of the possible role of HLA-B27 and ERAP1/2 in AS pathogenesis. HLA-B27 can present arthritogenic peptides to CD8+ T lymphocytes, which trigger AS initiation. Peptides enter the ER and are further trimmed by ERAP1 and ERAP2. Unusual peptides will be produced because of incorrect ERAP1 or ERAP2 trimming, leading to HLA-B27 free heavy chains (FHCs) and homodimers through endosomal recycling from the cell membrane and then to NK cell and Th17 cell activation by KIRs, particularly KIR3DL2. Abnormal peptide-HLA-B27 complexes gather in the ER, triggering UPR, ER stress, ER-associated protein degradation (ERAD) and autophagy. (Reprinted with permission of Elsevier, from Zohreh and colleagues^110^)

ERAP1, first reported in 2007^93^ as a risk factor for AS, is now considered the second strongest gene associated with AS; together with HLA-B27, ERAP1 is accountable for 70% of genetic factors for familial AS.^98^ It has been found that ERAP1 polymorphisms influence the risk of AS for HLA-B27+ individuals.^99^ Considering its function, ERAP1 may act in association with molecules such as HLA-B27 and be concerned with aberrant peptide processing or mistaken antigen presentation, leading to a predisposition to AS (Fig. 2).^99^ To date, five SNPs of ERAP1 have been recognized, i.e., rs27044, rs30187, rs2287987, rs10050860, and rs174820,^100^ and research has shown robust epistasis among ERAP1 SNPs, such as rs30187, rs27044, and rs27037, and HLA-B27.^101^ Research has shown that loss-of-function polymorphisms in ERAP1 affect HLA-B27 free heavy chain expression and peptide dimerization or misfolding. This research also shows a larger amount of ERAP1 production in dendritic cells (DCs) in patients, implying that abnormal ERAP1 expression may count for AS development to some extent.^102,103^ ERAP2 can form a heterodimer and cleave peptides collaboratively with ERAP1. Moreover, the functional variants in ERAP2 have been identified as involved in AS development.^90^ For example, the ERAP2 SNP rs2248374 causes nonsense ERAP2 protein expression and gives rise to the reduced expression of MHC-I molecules on the cell surface of AS patients.^104^ These polymorphisms causing a decrease in ERAP1 activity as well as a deficiency in ERAP2 production have now been recognized as some kind of defensive variants against AS.^90^ Therefore, the suppression of ERAP1 and/or ERAP2 may have protective effects and a promising treatment for AS.

### IL-23/IL-17 pathway

**Fig. 3:**
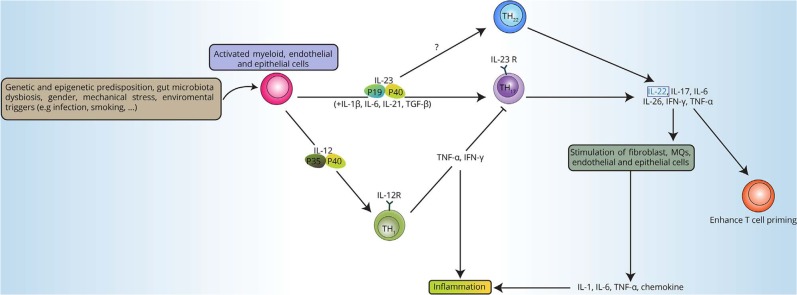
IL-23/17 pathway in AS pathogenesis. The interplay of genetic and epigenetic influences, particularly Th17 and Th22 cells, with a few kinds of stress, such as mechanical stress, gut microbiota stress, and environmental triggers, gives rise to the production of pro-inflammatory molecules, including IL-17, IL-22, TNF-α, and IL-23. (Reprinted with permission of Elsevier, from Zohreh and colleagues^110^)

In AS, the first indication of IL-23/IL-17 relevance came from a GWAS study in 2007 that identified an IL-23 receptor (IL-23R) SNP related to AS pathogenesis (Fig. 3).^34^ In humans, the differentiation of Th17 cells may be triggered by IL-23, TGF-β, and IL-1β, among other inflammatory cytokines, and the differentiated immunocytes further generate IL-17A, IL-17F, IL-22, IL-26, and CCL20.^105^ Dysfunction of the IL-23/IL-17 pathway was identified in many diseases related to human immunological procedures, including psoriasis, IBD, rheumatoid arthritis and SpA^106^; additionally, IL-17 and IL-23 act as major cytokines for axSpA and psoriatic arthritis.^107^ Studies have shown higher serum levels of IL-23 and IL-17^108^ and the presence of IL-17+ cells in the facet joints in AS patients,^109^ suggesting that the innate immune system might be of greater relevance. Moreover, it has been shown that AS can be greatly ameliorated by blocking the IL-23/IL-17 pathway,^105^ further indicating a significant role in AS development. In AS, differentiated T lymphocytes can generate IL-17 and then trigger osteoclast activation, thus suppressing bone regeneration. Moreover, lymphocytes can produce IL-22 when exposed to IL-23 to stimulate osteoproliferation.^110^ This contradictory process may explain the coexistence of erosion and formation of bone for patients with AS.

IL-23R genetic polymorphisms were considered related to AS vulnerability. The SNP rs11209026 leads to a nonsynonymous substitution of amino acid residue (R381Q) that considerably weakens IL-23R function, and the production of IL-17 can exert protective effects against AS inflammation and development.^111^ In addition to polymorphisms of the gene itself, variants in the vicinity of the IL12B locus, which encode the IL-12p40 subunit of IL-23, have been associated with AS.^112^ There are more IL-23/IL-17 pathway-associated loci, including IL-1R1, IL-2R, IL-6R, IL-12B, IL-27, STAT3, TYK2, CARD9, and RUNX3.^92^ A variant in the TYK2 locus is involved in AS pathogenesis by affecting TYK2 splicing with a strong odds ratio of 7.7.^90^ Based on these findings, it is reasonable to further concentrate on the connection of IL-23/IL-17 signaling and AS or possible therapeutic targeting molecules, including IL-1 and IL-23, and other downstream factors along the IL-23/IL-17 axis.

### Lymphocyte activation and differentiation

Another important non-MHC genetic factor in the pathogenesis of AS is genes modulating the activation and differentiation of either CD4+ or CD8+ T lymphocytes. GWASs of SNPs identified several non-MHC genes that are concerned with the production as well as activation of lymphocytes and connected to AS, including RUNX3, EOMES, ZMIZ1, IL7, TBX21, and IL7R.^90,112–114^ Runt-related transcription factor 3 (RUNX3), which belongs to the transcription factor family crucial for regulating the expression of lineage-specific genes, can stimulate T cell differentiation to CD8+ T lymphocytes in thymopoiesis.^115^ *RUNX3* polymorphisms have been linked to many human immune diseases or inflammatory processes, including systemic lupus erythematosus, psoriatic arthritis, and AS.^116,117^ Previous studies have revealed a connection between the *RUNX3* polymorphism rs11249215 and AS in Caucasian,^99^ Han Chinese^118^ and Korean populations,^119^ and rs4648889 has been related to reduced RUNX3 expression in AS.^120^ RUNX3 stimulates eomesodermin expression encoded by the EOMES gene and is the transcription factor related to the differentiation of CD8.^121^ In addition to RUNX3, research has suggested a relationship between these AS-associated non-MHC genes and lymphocyte differentiation or activation. A study of IL7R α chain knockout mice indicated that IL7R participation in T lymphocyte production can trigger RUNX3 expression for immature T lymphocytes and their differentiation towards the CD8 lineage.^122^ Polymorphisms of molecules involved in the activation or suppression of lymphocytes, such as programmed cell death 1 (*PDCD1*), encoding PD-1, or T lymphocyte antigen 4 (*CTLA-4*), encoding CTLA-4, have been shown to influence the susceptibility to AS.^106^ These non-MHC gene variants function in the pathogenesis of AS via unknown mechanisms, and further investigations are needed regarding their pathways and possible clinical applications.

### Immune cell and innate cytokines

**Fig. 4:**
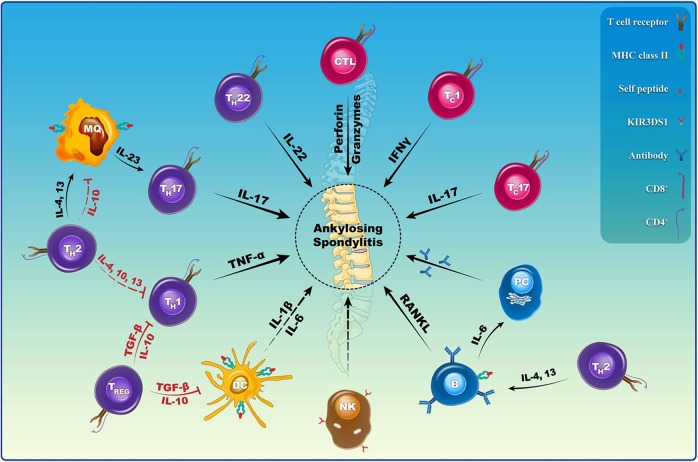
Immunocytes are involved in the initiation, evolution, and regulation of AS. (Reprinted with permission of Elsevier, from Mohammad and colleagues^123^)

AS is one type of seronegative spondyloarthritis, which is usually associated with chronic inflammation involving DCs, macrophages, NK cells and adaptive immune cells.^123^ These immune cells produce various innate cytokines that play a crucial role in the development of AS, as shown in Fig. 4. Human DCs located in lymphatic and nonlymphatic organs are divided into CD1c-positive (conventional DC1) or CD141-positive (conventional DC2) subsets.^124,125^ Another group of DCs, called plasmacytoid dendritic cells (pDCs), exhibit a plasma cell-like appearance and can produce CD56+, HLA-DR, derived dendritic cell antigen 2 (BDCA-2), Toll-like receptor 7 (TLR7), CD123, and TLR9 and can be distinguished from monocytes and conventional DCs by the lack of CD14 and CD11c expression.^126,127^ In addition to their function in the inborn and adaptive immune processes, these cells participate in B cell-mediated humoral immunity.^127^ Previous studies have shown elevated production of IL-1B and IL-6 in AS patients compared with that in normal subjects because a decrease in the number of circulating CD1c+ DCs increases the quantity of CD14-CD16+ mononuclear cells, which mediates the activation of CC chemokine receptor 6 (CCR6) expression.^124,128^ These processes trigger the Th17 immune response and IL-17 production, which are involved in autoimmune and inflammatory responses and are associated with clinical manifestations of AS.^129,130^ At the same time, CD1c-positive DCs can stimulate Th1 and Th2 responses. However, whether Th1 and Th17 cells act synergistically during the inflammatory process of AS is unclear. Some scholars believe that Th17 cells participate in the acute phase of inflammation,^131,132^ while Th1 cells function principally in prolonging or promoting late inflammatory reaction.^133,134^

The frequency or quantity of macrophages for AS subjects is closely related to disease severity.^130,135^ Large numbers of CD68-positive macrophages or osteoclasts were observed in the sacroiliac joint lesions of AS patients.^136^ Previous research has confirmed the major role of CD163+ macrophages in inflamed body parts for SpA patients.^137^ Moreover, the number of macrophages was reduced after drug therapy in SpA patients.^138^ A previous study using HLA-B27/human β2m transgenic rats has shown that HLA-B27+ macrophages produced IL-23-based inflammatory factors, which contributed to the pathogenesis of enthesopathy.^139^ In the clinic, the expression of IL-23 was significantly increased in synovium tissues and serum in AS cases compared to that in healthy controls.^20,140^ Another animal experiment showed that lowering the level of macrophages has a protective effect against AS.^141^ The NK cell number is also higher in AS patients. It has been reported that the increased levels of IL-8 and SDF-1 in AS patients increase the expression of the NK cell-inhibitory receptor carcino-embryonic antigen-cell adhesion molecule (CEACAM1), thereby inhibiting NK cell activity. NK cells specifically recognize HLA-B27 molecules through the NK-inhibitory receptor KIR3DL1. Thus, these two types of cells are important in chemokine expression and development of AS.^142^

A few subgroups of CD4+ T lymphocytes participate in the development of AS. Wang et al.^143^ found significantly higher levels of IFN-γ-inducible protein 10 (IP-10/CXCL10), attracting Th1 cells and even causing an increased level of IFN-γ and TNF-α and aggressive inflammatory responses. Moreover, the Th2 cell response could be enhanced in AS patients due to the overexpression of the chemokine receptor CCR4 on CD4+ T cells.^144^ Raised serum TRAC and MDC levels in AS subjects are positively related to the migration of Th2 cells.^143^ An elevated level of Th1 density as well as an increased Th1/Th2 fraction was detected for cases with mild or severe AS.^145^ These results suggest that the adaptive reaction is turned on to attract more Th2 cells and restore the Th1/Th2 ratio.^143^ Th17 cells, whose differentiating process is regulated via the transcription factor signal transducer plus activator of transcription 3 (STAT3) and STAT5, mainly produce pro-inflammatory cytokines, including IL-17, IL-10, IL-21, and IFN-γ.^146,147^ Research has revealed elevated serum IL-17 and IL-23 concentrations for AS patients.^148^ It has been reported that in predisposed patients, the IL-23/IL-23 receptor complex results in increased IL-17 production by inducing the proliferation and terminal differentiation of Th17 cells.^149^ The IL-23/IL-17 axis causes inflammatory molecule secretion from fibroblasts, endothelial cells, DCs or macrophages, which induce joint destruction and inflammation that are observed in both rheumatoid arthritis and AS.^140,146^ The quantity of Th22 cells in peripheral circulation and the IL-22 concentrations in plasma were shown to increase without an obvious relation to AS severity.^150^ El-Zayadi et al.^151^ found that IL-22 could drive the propagation, migration and osteogenic differentiation of mesenchymal stem cells and as such, might be a novel cytokine boosting bone construction in SpA. Nevertheless, the level of IL-22 producing CD4+CD25highCD127low/- regulatory T cells (Tregs) was meaningfully lower for AS cases than for controls, but it is unclear whether the level of Tregs could be considered a predictive factor for disease severity or treatment outcome.^152,153^

Zhang et al.^154^ found that mononuclear cells in peripheral circulation in AS and HLA-B27-positive normal cohorts showed an elevated level of IL4+CD8+ T cells compared to those from HLA-B27-negative control cohorts, accompanied by an increased proportion of IL-4+ to IFN-γ+ cells. Previous studies have described that HLA-B27-restricted T cells could be responsive to self-antigens and arthritis-implicated antigens, resulting in autoimmune inflammation.^155,156^ Researchers have found that CD8+ T cells could respond to peptides LMP2 236-244 and VIP1R 400-408 for HLA-B2705 and HLA-B2709 individuals via this pathomechanism.^157^ CD8+ T cells could cause the direct lysis of target cells by CTLs via the secretion of perforin/granzyme or Fas/FasL signaling. These cells could also generate inflammatory products, including TNF-α, IFN-γ, and IL-17, to maintain chronic immune reactions for AS patients.^158,159^

Studies on B cells in AS have shown different results from those on T cells. An elevated number of plasma cells and plasma blasts has been found in the circulation and peripheral joints of AS patients.^160,161^ Based on current knowledge, B cells function as effectors possibly involved in the pathogenesis of AS through various mechanisms. First, B lymphocytes can differentiate into plasma cells that secrete antibodies and affect immune reactions, even triggering osteoclast genesis. Second, B lymphocytes produce cytokines such as IL-6 and receptor activator of nuclear factor kappa-B ligand (RANKL), which stimulate plasma cell formation and osteoclast genesis separately. Moreover, B lymphocytes can present antigens, further serving as costimulators during suitable activation of T lymphocytes. Last but not least, B lymphocytes help in forming germinal center-like ectopic lymphoid tissue for plasma cell generation.^162^

In conclusion, although multiple autoantibodies including anti-CD74 and anti-NOG/SOST have been found, the role for B cells is quite limited, and AS is still identified as a seronegative arthritis.

## Treatments for AS

### Pharmacological treatments

**Fig. 5:**
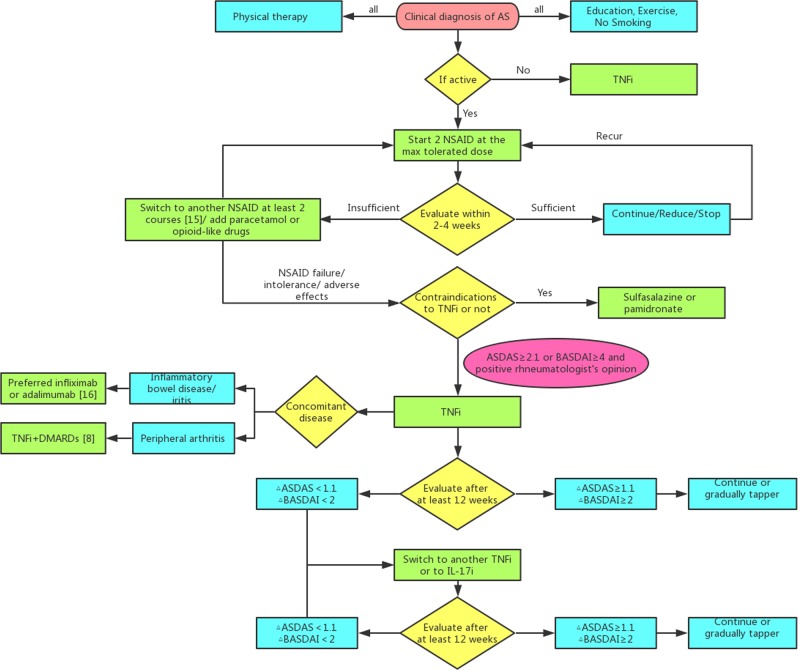
Drug treatment strategy for ankylosing spondylitis patients

The aims of treating AS are to improve and maintain spinal flexibility and normal posture, relieve symptoms, decrease functional limitations, and reduce complications. The mainstays of pharmacological treatment involve nonsteroidal anti-inflammatory medications (NSAIDs) and TNF-α inhibitors (TNFis). Additional treatments include non-TNFi biologics (secukinumab), methotrexate, and sulfasalazine. Furthermore, the oral small molecule JAK inhibitors tofacitinib and filgotinib appear promising in clinical trials and may soon be approved for AS.^163,164^ Several guidelines for the management of AS have been issued by expert panels in France,^165^ Spain,^166^ Tukey,^167^ Canada,^168,169^ the United Kingdom,^170^ the United States^171^ and Europe,^172^ which are all based on systematic literature reviews. As shown in Fig. 5, there is substantial agreement among these strategies. For all AS patients, regardless of whether the disease is active or stable, physical therapy,^173^ exercise and abstaining from smoking^174^ are universally advised.

NSAIDs, especially selective inhibitors of cyclooxygenase 2, are first-line treatments for patients with active AS. The determination of active disease is founded on laboratory (CRP/ESR), clinical and imaging (magnetic resonance imaging, MRI) findings.^175^ Compared with on-demand treatment, continuous NSAID treatment has shown no benefits in any clinical aspect,^176^ while hypertension and depression are more common among individuals undergoing continuous NSAID treatment. However, continuous use should be advised if symptom recurrence occurs after stopping or reducing the dose of NSAID drug.^177,178^ In adults with active AS, an appropriate trial consists of at least 2 kinds of NSAIDs, each administered over a minimum of 2 weeks at the maximum tolerated dosage, unless contraindicated.^179^ Nevertheless, the ‘lowest effective dose’ of NSAIDs has also been recommended in the National Institute for Health and Care Excellence (NICE) guidelines.^170^ No NSAID is recommended in terms of preferred efficacy. NSAID treatment should be chosen based on the patient’s history of NSAID application, comorbidities, and risk factors for adverse effects.^171^ Good responses to NSAIDs include a reduction in inflammatory back pain and functional improvement. An insufficient response to NSAID therapy is identified as active disease despite the administration of at least two different NSAIDs at the maximum anti-inflammatory dose and duration (at least two weeks for each). Intolerance or adverse effects are also involved. Analgesics, especially opioid-like drugs, may be added when NSAID treatment is unsuccessful or contraindicated.^167,172^

Despite treatment with NSAIDs, TNFis are advised for patients with high disease activity.^171^ An insufficient response to TNFis is defined as disease activity markers remaining over specific cut-offs (BASDAI ≥ 4 or AS Disease Activity Score (ASDAS) ≥ 2.1) over time. The ASDAS reflects the inflammatory condition better than the BASDAI, and its cut-offs are preferable. Biological agents should be used according to their indications and contraindications and patient comorbidities. No TNFi is recommended for preferred efficacy. Moreover, treatment with infliximab or adalimumab is preferred over treatment with etanercept for patients with IBD or frequently recurrent iritis.^180,181^ Predictors for a good response to TNFis are a short disease duration, patient age ≤40 years, the absence of enthesitis, positivity for HLA-B27, a good functional status, and a high CRP level.^182^ In a Swiss study, male sex was also identified as a good predictor for TNF response.^183^ Contraindications for TNFis include the presence of an active infection, tuberculosis, advanced heart failure, lupus, multiple sclerosis and cancer. In patients with active AS and who have contraindications for TNFis, sulfasalazine or pamidronate is recommended over non-TNFi biologics, such as abatacept and tocilizumab.^171^ When AS patients fail to respond to the first TNFi, treatment with a second biologic should be advised. The different biologics can be an IL-17 inhibitor (IL-17i) or a different TNFi.^184^ The treatment should be changed if no significant improvement occurs after application for 3 months. If a 6-month trial results in no clinical remission or decrease in disease severity, the treatment must be changed. After failure treatment of the first TNFi, a second TNFi with a lower efficacy can also be effective.^184^ However, before switching the treatment, it is essential to reconsider the indications of the first TNFi. Given the circumstances of a primary lack of efficacy, primary failure might be due to an incorrect diagnosis rather than drug resistance. Furthermore, the symptoms and signs may result from either a different or concomitant condition. Biologics may fail in AS patients with concomitant vertebral fracture or degenerative disc disease. In patients with persistent remission, the tapering of TNFi or IL-17i treatment can be considered.^185^ The period of remission should be at least 6 months. Ideally, tapering can be continued to zero (withdrawal). However, tapering only very slowly and allowing sufficient time for remission are suggested before the next step in the tapering process.

Local injections of glucocorticoids seem to be an option for treating enthesopathy and arthritis. Glucocorticoid injections into involved peripheral joints, sacroiliac joints, or entheses could provide immediate symptom relief. Previous studies have shown that partly because of the increased risks of osteoporosis, hyperlipidemia and insulin resistance, long-term treatment with systemic glucocorticoids is relatively contraindicated. A recent study reported that AS patients achieved relief from signs and symptoms after short-term treatment with high doses of glucocorticoids (50 mg/day).^186^ In patients with peripheral arthritis as a comorbidity, conventional synthetic disease-modifying antirheumatic drugs (csDMARDs), such as methotrexate, leflunomide, and sulfasalazine, should be considered but not in those with isolated axSpA or enthesitis.^172^ Methotrexate treatment has not been proven effective in AS patients without peripheral arthritis, regardless of administration of a TNFi.

Other biologics include rituximab (a monoclonal antibody against CD20+ B cells), ustekinumab (a monoclonal antibody against IL-12/23) and secukinumab (a monoclonal antibody against IL-17). If TNFi fails to treat AS, rituximab may be an alternative approach.^187^ In AS patients with concomitant moderate to severe psoriasis, ustekinumab treatment reportedly achieved safe and significant improvements.^188^ Ustekinumab can also reduce arthritis, enthesitis, dactylitis, and skin lesions and improve function.^189^ In a prospective clinical trial, ustekinumab was associated with a reduction in signs and symptoms in active AS and was well tolerated.^190^ However, a recent study did not demonstrate the efficacy of ustekinumab (anti-IL12p40) and risankizumab (anti-IL23p19) in the treatment of axial SpA.^191,192^ At the moment, the mechanism of blocking IL-23, which does not play a role in SpA treatment, remains uncovered.^193^ IL-17A and IL-22 could be suppressed in anti-IL23R in prophylactic experiments^194^ and were comparable to those after therapeutic anti-IL23R treatment. Thus, the initiation instead of the persistence of experimental SpA may depend on IL-23 signaling. Secukinumab targets IL-17 and is effective in patients with TNFi failure.^195^ In a phase 3 trial, secukinumab showed salient efficacy in AS patients with an insufficient response or contraindications to TNFis.^196^ In another phase 3 trial, secukinumab was verified to provide sustained efficacy in signs, symptoms and physical function in subjects with AS over 3 years.^197^ A study cohort of Taiwanese patients indicated that secukinumab was also well tolerated in Asian patients, with a safety profile consistent with that reported in the overall study population.^198^

In patients with stable AS, using NSAID treatment on-demand is recommended. Continuing treatment with TNFi alone is suggested rather than treatment with TNFi and NSAID or DMARD.^199^ The continued use of NSAIDs or DMARDs has uncertain therapeutic effects with increased risks of gastrointestinal, cardiovascular, renal and hematological toxicity.^200^

### Surgical treatments

Untreated AS can cause spinal deformity, with more than 30% of AS patients suffering from thoracolumbar kyphosis.^201^ Corrective osteotomy and stabilization are very common in surgical procedures and are recommended under certain conditions,^171^ such as adult patients suffering severe kyphosis or advanced hip arthritis. This procedure has a perioperative mortality rate of 4% and permanent neurologic sequelae rate of 5%.^202^ This surgery is confirmed to contribute to preventing the natural processes of progressive deformity, reducing pain caused by muscle fatigue, improving disability, restoring the global balance and horizontal axis of view, and improving respiratory and digestion function.^203–210^

Osteotomy procedures for the treatment of kyphotic deformity can be fundamentally differentiated into closing- vs. opening-wedge osteotomy (CWO/OWO) procedures.^210^ In 1945, OWO was first introduced by Smith-Petersen et al.^211^ and has subsequently been modified by several surgeons.^212,213^ For correction of the kyphotic deformity of the lumbar spine, this technique creates a gap in laminae and spinous processes, forcing manual extension of the lumbar spine. The complication rate is relatively high due to the sharp lordotic angle and the elongation of the anterior column occurring in this procedure, which can cause serious vascular and neurological injuries.^214,215^ To solve this problem, Wilson et al.^216^ first presented poly-segmental wedge osteotomy (PWO) in 1949, which was improved by Zielke in the 1980s.^217–220^ Correction was achieved via multiple CWOs in the posterior lumbar spine to generate a rather harmonious opening of the anterior disc spaces with tempered posterior shortening spanning. Zielke and his coworkers, different from the former procedure, advocated PWO with internal fixation using Harrington rods, laminar hooks, and later transpedicular screws. Nevertheless, on the instrumentation, implant failure has been described in >40% of patients due to limited mobility of the intervertebral disc and strong effect.^221^ Thus, the postoperative satisfaction rates were far from the expected rates.^221–223^

In 1963, monosegmental CWO was first introduced by Scudese and Calabro^224^ and thoroughly developed by Ziwjan in 1982^225^ and Thomasen in 1985.^226^ During this procedure, the posterior elements of one vertebra, in combination with the posterior wedge of the vertebral body, are resected to achieve correction by passive extension of the lumbar spine. Similar to PWO, internal fixation is also needed to enhance immediate stability. In this technique, correction is achieved. However, the postoperative satisfaction and complication rates of CWO seem to be better than those of OWO or PWO.^226,227^

Based on these three surgical procedures, surgical techniques for treating kyphosis have been constantly improved. Closing–opening-wedge osteotomy (COWO), particularly beneficial in cervical spine surgery, was first introduced by Kawahara et al.^228^ to overcome some limitations and simultaneously combine the benefits of CWO and OWO. Posterior column resection is performed in a manner similar to that of CWO, with the tip of the wedge at the vertebral midsagittal point. A plane is osteotomized anteriorly from this point parallel to the endplates or the anterior cortex is fractured and the anterior column is opened during osteotomy closure.^210^ This procedure has been shown to reduce localized kyphosis from an average of 67–18° at 2.2- to 7.5-year follow-ups.^228^ Ji et al.^206^ and Bourghli et al.^229^ have shown similar results; in addition, spinal cord shortening and aorta lengthening were well tolerated in all patients.

Regarding the incidence of spine fractures, it is estimated to be 4 times greater in patients with AS than in the general population,^230^ largely because of the combination of rigidity and osteoporosis that develop in these patients.^231^ Spine fracture can be severe even after minor trauma^232,233^; 75% of these fractures occur in the cervical spine, particularly at the C5-T1 cervicothoracic junction.^234,235^ Because these fractures often follow minor trauma (47%)^236^ and occur in patients who typically already have long-standing back pain, the diagnosis is often delayed. To make matters worse, these fractures can be missed on X-rays and often require CT for diagnosis.^237^ Neurological deterioration during the initial hospitalization after spinal fractures in the context of AS is common, and the 1-year mortality rate is high.^238^ There is a consensus on the optimal therapeutic treatment of spine fractures in patients with AS. Most reports in the literature describing the surgical treatment of spine fracture in AS patients have been limited to case reports and smaller series.^239,240^ Complete spinal cord paralysis persisting for more than a few hours does not seem to benefit from surgery.^241^ A retrospective study conducted by Backhaus *et al*.^236^ included 119 patients with 129 spine fractures due to AS. Sixty-one patients (51%) developed either incomplete or complete paraplegia. Furthermore, revision surgery due to implant loosening or insufficient stabilization was required in 15% of patients. For some patients, the risks would outweigh the benefits. Therefore, evidence for routine surgical interventions is limited. For patients with threatening or definite neurological deficiencies, surgery may be considered by experienced surgeons.

Cauda equina syndrome (CES) is a rare complication of long-standing AS. Neurological symptoms occur insidiously and have a poor prognosis without effective treatment. AS-related CES may be due to chronic arachnoiditis and dural fibrosis leading to diminished cerebrospinal fluid (CSF) resorption with dural sac dilation and diverticula formation.^68,242^ Leaving these patients untreated or treated with steroids alone is inappropriate. Surgical treatment of dural ectasia, either by lumboperitoneal shunting or laminectomy, may improve neurological dysfunction or halt the progression of neurological defects.^243^

Hip abnormality is commonly recognized by rheumatologists in AS patients and affects approximately one-fourth to one-third of AS patients.^244^ Proposed by the ACR, based on evidence, arthroplasty is the best treatment option for patients with advanced hip arthritis and severe hip pain who often would otherwise experience progressive limitations in mobility and reliance on opiates.^171^ The annual trends of total hip arthroplasty (THA) in AS patients have significantly declined, which may be attributed to improvements in medical management that delay the time from disease onset to THA.^245^

There have been many studies on THA, although THA could be a difficult procedure in patients with AS. Based on current reports, THA can generate satisfactory results, greatly improving hip joint function and relieving pain without significant complications.^246–250^ Data from 54 AS patients who underwent 81 THAs between 2008 and 2014 were retrospectively analyzed by Xu et al.^246^ Significant improvements in the Harris hip score (HHS), visual analog scale (VAS) score, and range of motion (ROM) were found postoperatively, with favorable prosthetic localization observed by radiographic evaluations; all of these findings indicated acceptable short- and mid-term benefits. A total of 181 hips of 103 patients were included in another large study with a follow-up period of over 27 years conducted by Joshi et al.^251^ A satisfactory survival rate (71%) and no functional impairment or reankylosis were reported, demonstrating the long-term improvement of hip function for AS patients.

Nevertheless, evidence for the efficacy of THA in AS patients is based only on observational studies and case series. Controversies persist concerning the surgical timing, implant selection, intraoperative management strategies, and heterotopic ossification (HO) prophylaxis. Concomitant severe hip and spinal deformity is particularly challenging to treat, and there is no consensus on which deformity to repair first.^246,252–256^ Prior to THA, some people advocate that a spinal osteotomy should be performed to reduce the risk of hip dislocation.^246,253–255^ Others agree with the assertion that a THA performed first would contribute to placement of the patient in a stable prone position to facilitate corrective spine osteotomy.^256^ Another debate has been focused on the superiority of cementless or cemented components in AS patients. Cemented prosthetic components are recommended for AS patients with severe osteoporosis who should be treated with revised total hip replacement (THR) to ensure a good fit of the prosthesis to the canal.^251,253,257^ Additionally, the failure rate of THR using cemented components (5%) is lower than that of THR with cementless components (28%).^253^ In contrast, cementless prostheses are preferred in AS patients, especially young patients, without significant morphological changes in the proximal femurs because this strategy can reduce the difficulty of future revisions while allowing bone ingrowth to enhance the durability of the implant.^258,259^

HO is the main postoperative complication of joint arthroplasty, with high occurrence rates ranging between 4 and 74.7%.^253^ There are no clear recommendations on perioperative precautionary measures for HO after THR in AS patients. Previous studies have suggested the use of NSAIDs, such as indomethacin, and radiation therapy, both preoperatively and postoperatively, to prevent HO,^253,258,260,261^ while others believed that the risk of prevention outweighed the benefits.^246,251,262^ To minimize the adverse effects, Feng et al.^248^ shortened the medication time by application of indomethacin for 2 weeks as prophylaxis. No patients who received perioperative prophylaxis developed reankylosis by HO in this study.^248^ Although radiation therapy has been proven effective for preventing HO, another retrospective study including 129 hips of 91 Asian AS patients conducted by Weng et al.^263^ showed no significant difference between the postoperative single-fraction radiotherapy group and the control group (*P* = 0.210), suggesting that postoperative radiation may not be necessary in Asian patients.

## Conclusions and perspectives

Ankylosing spondylitis is a painful and debilitating disease, with considerable socioeconomic burdens. In 2002, the yearly mean total (direct and productivity) costs of AS were US$6,720 per patient in the USA and Euro 9 462 per patient in Europe.^264^ There is no effective disease-modifying treatment largely due to the unclear pathogenesis. This review focuses on the etiology, pathogenesis and treatment progress of AS. The etiology of ankylosing spondylitis may be related to genetic background, immune function, pathogenic infection, endocrine abnormalities and other factors. HLA-B27 is the gene crucial to the development of ankylosing spondylitis, while the non-HLA-B27 gene is also important in its pathogenesis. Genetic differences between various ethnicities, particularly East Asian and Caucasian descent, have been noted through GWASs and may shed light on therapeutic strategies. Certain autoimmune diseases and microbial infections also contribute to ankylosing spondylitis, suggesting that they may have a common immune and genetic basis. In addition, the possible causes include hormone abnormalities and changes in vitamin D levels.

The pathogenesis of AS is very complex. Current studies suggest that it may be the result of a variety of complicated mechanisms. In the pathogenesis of AS, various hypotheses, such as the false folding hypothesis, attempt to elucidate the role of HLA-B27. Other non-HLA-B27 genes may influence the occurrence of ankylosing spondylitis through immune function and gene interaction. Overexpression of ERAP1 and ERAP2 is also considered an important pathogenesis of AS. Many factors and related genes in this pathway are constantly being discovered. In addition, related research has focused on the disorder of the IL-23/IL-17 axis and the abnormality of lymphocyte activation and differentiation. Most immune cells and cytokines reportedly participate in the pathogenesis of AS. In particular, the IL-23/IL-17 pathway plays a crucial role in the development of the disease. At present, the pathogenesis of AS is considered to mainly involve immune T cells, while B cells are also slightly involved. There are some studies on the pathogenesis of AS mediated by B cells, and related studies may be strengthened in the future. Alternatively, researchers may continue to explore the correlation among cytokines and immune cells and diseases to predict the occurrence, development and severity of disease. Although the pathogenesis of ankylosing spondylitis is not yet clear, the existing research results can have a certain guiding significance for clinical practice.

The treatment of AS is mainly composed of drug and surgical treatment. In clinic, NSAIDs and TNF-alpha inhibitors are the main drugs for AS. Moreover, interleukin receptor blockers and some drugs that inhibit new bone formation have received increased attention and become the focus of future research, including IL-6 receptor inhibitor sarilumab and Wnt signal pathway inhibitors. Once AS is not effectively controlled, more severe deformities may appear, and surgical treatment is needed. For severe spinal deformities, spinal surgery is required. For sacroiliac joint lesions, total hip arthroplasty is needed. Treatment of AS has always been the center of research all over the world. Currently, the research hotspot of surgical treatment of AS focuses on the choice of surgical path, perioperative management, and the prevention and treatment of complications, especially HO. With scientific and technological development, early diagnosis and effective treatment of AS will be possible. The socioeconomic burden of AS will be significantly reduced, and patients' suffering will be alleviated.
